# Persistence of pain in patients with chronic low back pain reported via weekly automated text messages over one year

**DOI:** 10.1186/s12891-015-0754-4

**Published:** 2015-10-15

**Authors:** Charlotte Leboeuf-Yde, Rikke Krüger Jensen, Niels Wedderkopp

**Affiliations:** Research Department, Spine Centre of Southern Denmark, Hospital Lillebaelt, Østre Hougvej 55, DK- 5500 Middelfart, Denmark; Institute of Regional Health Services Research, University of Southern Denmark, Middelfart, Denmark; Sports Medicine Clinic, Orthopaedic Department, Hospital Lillebælt, Østre Hougvej 55, DK-5500 Middelfart, Denmark

**Keywords:** Non-specific low back pain, Bothersomeness, Modic changes, Persistent, Chronic, Text-messages

## Abstract

**Background:**

A previous study has suggested that it is uncommon for patients with chronic bothersome low back pain (LBP), who consult the secondary health care sector, to report at least four consecutive weeks without such bothersome pain in 1 year. It is not yet known, however, how many days of the week they experience pain throughout the year.

**Method:**

The current study analyzed data collected in two randomized clinical studies conducted in 2007–9 on patients with back pain (Study 1 and 2). Study participants were patients with LBP for more than 2 months, one group with MRI-defined Modic changes (Study 1) and the other without any pathological explanation for the pain (Study 2). In both studies, participants were followed over 1 year with weekly automated text messages (SMS-Track). Each week they reported the number of days they had experienced bothersome LBP (0–7 days). The number of weeks with 7 days of bothersome LBP was calculated for both study groups. As baseline and outcome characteristics were similar between the intervention and control groups in each study, the data from treatment and control groups in each study were analyzed together, regardless of treatment allocation and the results compared between the two study samples.

**Results:**

The proportion of patients reporting bothersome LBP all days of the week ranged from 0 to 100 %, with the findings arranged in a U-shaped curve. The pain frequency patterns were remarkably similar for the two study samples. At one extreme, 31 % of participants reported 0–10 % of weeks with daily LBP. At the other extreme, 25 % of participants reported 91–100 % of weeks with daily LBP. The distribution between these values was also very similar for the two groups.

**Conclusion:**

This study revealed there to be considerable variation in weekly persistence of symptoms during 1 year in patients from the secondary care sector with chronic LBP. The results range from bothersome pain each day of the week, every week of the year, to no weeks at all with 7 days of pain. Interestingly, this pattern is near-identical in the two study samples; those with non-specific LBP and those with LBP and Modic changes. This heterogeneic pain profile in patients with chronic LBP deserves to be further investigated.

## Background

Low back pain (LBP) is common in the general population. In a large Danish study that included 34,902 individuals aged 20–71 years, 12 % reported having LBP for at least 30 days in total during the preceding year with no obvious variations in relation to age and sex [1]. However, in a smaller study of the general middle-aged Danish population, in which data were collected every fortnight over 1 year, approximately one-third of respondents could be classified as having chronic “bothersome” LBP [2].

Regardless the exact definition of LBP, the proportion of respondents with chronic or very persistent LBP is likely to differ depending on the origin of the study sample. We would thus expect a step-wise increase going from the general population (via the primary care sector) to the secondary care sector. One would also expect that different LBP-groups would be differently affected, such as people with more benign diagnoses vs. those with definite, pathological labels.

Although information is available in the literature on the outcome of LBP at different points in time both in the general [3] and clinical [4] populations, no study appears to have compared the trajectory over time in different types of clinical populations, a method that would bring more detailed information than simple retrospective information on self-reported duration of pain in the past.

Two clinical study populations with chronic LBP have previously been followed over one year with automated text messages (SMS-Track) [5]. Study participants were patients at a specialized outpatient spine clinic who were subsequently included in one of two randomized controlled clinical trials, one dealing with patients with LBP and Modic changes and the other including patients with LBP of unknown origin, so-called non-specific LBP. During the study period of 1 year, participants were asked every week, how many days in the previous week that they had “problems with LBP” (translated from Danish). For ease of reporting this will be referred to as “bothersome LBP”, as the term “bothersome LBP” is a commonly used term for LBP that exceeds the occasional twinge or simple awareness of some pain, and as the term correlates with the conception of the original phrasing in Danish.

A previous analysis of these two study samples [5] showed that the majority of participants (65 and 56 %, respectively) never reported an entire week without bothersome LBP during that year. The proportion who at some time during the study period reported 0 days of bothersome LBP for at least 4 weeks in a row was 20 and 18 %, respectively. A four-week duration period was used, as it was previously recommended as a suitable period of demarcation for absence of LBP for research purposes [6].

Because absence of bothersome LBP was so rare, a preliminary conclusion could be that these two groups of patients have true chronic LBP. However, even though it was more common to report weeks with at least some bothersome LBP than weeks entirely without, and even though absence of bothersome LBP for an entire month was never reached by 80 % of the participants, there would still be room for considerable variation during the weeks, when at least some bothersome pain could be reported but not necessarily each and every day.

To establish the exact extent of truly persistent bothersome LBP, it would be relevant also to determine the number of weeks that study participants reported such LBP every day. We returned, therefore, to our previous data material from these two clinical study samples [5], this time to study the one-year pattern for weeks with 7 days of bothersome LBP and not, as in the previous report, the one-year prevalence of weeks with 0 days of pain. Baseline and outcome variables were similar for participants in the intervention and control groups in both studies. For the purposes of the current SMS-Track study, therefore, data were combined and analyzed together for each study regardless of treatment allocation, as it was done in the previous study.

## Method

### Original studies, study participants and ethics permission

Data were available from two randomized clinical trials (Study 1 and Study 2) carried out during 2007–9 as part of PhD projects. Results have been reported elsewhere for one of the studies [7], but the other study has remained unpublished. The profile of the study participants and information on missing data, have been reported in detail elsewhere [5].

Participants were aged 18–60 years and had been referred from general practitioners or chiropractors to a Danish outpatient spine clinic specializing in back pain. Those who fulfilled some minimal criteria (LBP or leg pain ≥3 out of 11 on a numerical rating scale, and a duration of 2–12 months), were screened with a baseline questionnaire and magnetic resonance imaging (MRI).

Patients originally invited to be included in Study 1 (*N* = 100) had localized LBP and any type of Modic changes that extended beyond the vertebral endplate, as defined by MRI. Treatment consisted of either a 10-week exercise program based on the “do not worry-keep active” concept or instructions to rest and refrain from hard work.

Participants in Study 2 (*N* = 258) were patients who were assumed to be suffering from non-specific LBP as they did not have an obvious acute disc problem or other pathology, and were not eligible for Study 1. The intervention group received a needs-based psychosocial approach in addition to the “usual” treatment provided to the control group that included physical examination, information, treatment in the form of training, spinal manipulation and/or medication as considered relevant by the treating clinician, and advice on continued management.

Baseline characteristics were similar for the intervention and control groups in each study. Thus in Study 1, the two groups were similar in terms of age, sex, body mass index, smoking, type of occupation, education, sick leave, LBP intensity, activity limitation, general health, and symptoms of depression. In Study 2, the two groups were similar in terms of age, sex, educational level, psychosocial profile, previous LBP and LBP intensity, dispute about work accidents, and activity limitation. Furthermore, outcomes and number of drop-outs were similar for the intervention and control groups in each study. For the purposes of the current study, therefore, the text message data have been combined for the intervention and control groups in each study, resulting in two samples for statistical analysis (one consisting of all participants in Study 1 and the other of all participants in Study 2).

Patients received written and verbal information about the study, signed informed consent forms and were allowed to withdraw from the study at any time without any consequences for their future treatment at the centre. Both studies were approved by the Ethics Committee for the Region of Southern Denmark (# S-VF-20060111 and 12165) and were registered in the ClinicalTrials.gov data base (Identification numbers NCT00454792 and NCT00459433). Permission for this additional analysis was given by the Research Department.

### Data collection and storage

In the original studies, patients completed baseline and follow-up questionnaires and were also followed over 1 year with weekly automated text messages (SMS-Track. http://www.sms-track.com). This report deals only with the SMS-Track data. Each week participants were asked, via a text message, how many days in the previous week they had experienced bothersome LBP. They answered by typing in the relevant number, e.g. 0 for no LBP, or three for 3 days of LBP. Their answers were automatically entered into a data file.

In Study 1, at the onset of the study, phone calls were made to people who failed to respond in order to provide extra explanation of how to respond to the text messages and to improve the compliance. This procedure was not used in Study 2.

### Variable of interest and analysis of data

The current analysis used only one of the LBP variables that were collected. The item had the same wording in Study 1 and Study 2: “Using a number between 0 and 7, please answer how many days in the past week you have had problems with your lower back.”

Data were collected over 53 weeks. Out of the total number of weeks that a participant returned text messages, we calculated the percentage of weeks that the participants reported bothersome LBP for all seven days. We excluded participants who had none or only occasional responses during the study period. Some participants whose data sets were included in the analysis also had some missing data, but we decided against using imputation or worst/best case analysis. Instead, a separate analysis was made on study subjects who participated for less than 50 % of the weeks, to see if their profile was different from that of the others. No attempts were made to cluster findings on any other type of information.

The results are reported as percentages of weeks with 7 days of bothersome LBP and 95 % confidence intervals. Estimates were near-identical between the two studies, so it was not relevant to test for statistically significant differences.

### User-friendliness and validity of text messaging data

A previous inter-method reliability study on participants in Study 2 showed that weekly text messages sent over 1 year were acceptable to participants, and that retrospective data would be more affected by memory decay than frequent data collection [8]. Another previous study on patients with LBP showed the SMS-Track system to be user-friendly, yielding high response rates unaffected by season, and the few drop-outs were not predominantly young men, as is often seen in studies with multiple follow-ups and long follow-up time [9].

## Results

### Missing data

In Study 1, 20 of the 100 participants dropped out early during the intervention and were therefore not followed with SMS-Track afterwards. The remaining 80 study subjects had only occasional missing data. In Study 2, however, 19 data sets were removed, as they provided too little information. Of these, 16 participants never responded to any SMS-Track messages and the other three answered only once, twice or three times. Of the remaining 239 participants, 30 provided an answer less than 50 % of the time and were analyzed separately, leaving 209 individuals for the main analysis. For a detailed account of the number of missing weeks in the two study samples used for analysis, please see Table 1.

**Table 1:** Number of weeks with missing text-message answers over 1 year in two samples (Study 1; *N* = 80 and Study 2; *N* = 209) used for analysis of bothersome low back pain

Number of weeks with missing text-message answers	Number of individuals in Study 1 who failed to answer	Number of individuals in Study 2 who failed to answer
0	58	83
1–5	17	84
6–10	3	15
11–15	1	6
16–20	0	13
21–26	1	8
Total	80	209

### Descriptive data

Analysis was performed on data from 80 participants in Study 1 (mean age 46 years, range 21–61 years, 68 % women) and 209 participants in Study 2 (mean age 38 years, range 18–60 years, 54 % women). At baseline, mean level of severity of LBP was rated at 5.3 (Study 1) and 4.9 (Study 2) on a 0–10 scale.

### Prevalence of weeks with seven days of bothersome LBP

**Fig. 1:**
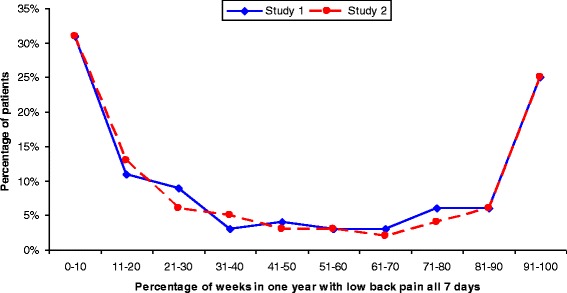
Percentage of weeks with 7 days of bothersome low back pain reported by a) 80 patients with low back pain and Modic changes (Study 1) and b) 209 patients with non-specific low back pain (Study 2) collecting data every week with SMS-Track over 1 year. At the *left* extremity of this figure, categories with 0 % and 1–10 % have been merged as have categories 91–99 % and 100 % at the *right* extremity

The results from both groups revealed a U-shaped curve for weeks with 7 days of bothersome LBP, see Fig. 1. At one extreme, 0–10 % of weeks consisting of bothersome LBP each day of the week was reported by 31 % of participants in both studies. At the other extreme, 91–100 % of weeks of pain each day of the week were reported by 25 % in both studies. The spread of data between these values was also very similar between the two groups (Table 2, columns 2 and 3). The participants in Study 2 who provided an answer less than 50 % of the time had a similar profile to the others (Table 2, last column). The main minor exceptions were that somewhat more of the poor responders reported ≤10 % of pain days of the others (40% vs. 31 %), and that they were somewhat less likely to report pain days during at least 91 % of weeks (20 % vs.25 %).

**Table 2:** Percentage of weeks with 7 days of bothersome low back pain reported by participants a) with low back pain and Modic changes (Study 1), b) with non-specific low back pain (Study 2), and c) participants in Study 2 who provided text messages less than 50 % of the time

Percentage of weeks in 1 year where LBP was reported to have been present all 7 days	Study 1: Patients with low back pain and Modic changes	Study 2: Patients with non-specific low back pain	Study 2: Patients who provided text messages less than 50 % of the time
	(*N* = 80)	(*N* = 209)	(*N* = 30)
%	%	%	%
0	16	10	30
1–10	15	21	10
11–20	11	13	3
21–30	9	6	10
31–40	3	5	10
41–50	4	3	10
51–60	3	3	3
61–70	3	2	-
71–80	6	4	-
81–90	6	6	3
91–99	15	10	3
100	10	15	17

## Discussion

This study appears to have been the first to have attempted to determine the extent of truly persistent bothersome LBP, through an analysis of the number of weeks in 1 year that patients with LBP report seven consecutive days of bothersome pain. The distribution of full weeks with bothersome LBP was U-shaped for the two patient groups, one with MRI-defined Modic changes and the other without any pathological explanation for the pain. Thus 31 % of participants reported 0–10 % of weeks with bothersome LBP each day, while 25 % of participants reported 91–100 % of weeks of pain. In fact, the spread of data was almost identical between the two groups both in relation to none, few, some, many, and all weeks over 1 year with 7 days of bothersome LBP.

These results contribute to current knowledge about the “true” persistence of LBP in patients with chronic LBP. A previous study showed that only a small minority of participants experienced a period free of bothersome LBP of at least four consecutive weeks [5]. Surprisingly, in the current analysis, the proportion with truly persistent bothersome LBP was also small, but the pattern of weeks with 7 days of pain “in between” showed considerable variation.

As found previously [5], the pain profiles of our two study populations were similar to each other, possibly indicating that the pattern of LBP is very similar regardless of etiology, or that the supposed etiology (Modic changes in Study 1, non-specific LBP in Study 2) is without relevance for this type of pain pattern. Interestingly, the baseline severity of LBP, measured on a 0–10 numerical rating scale, was 5.3 and 4.9 in Study 1 and Study 2, respectively, indicating that these two groups were also very similar at baseline and not only during the course of the study period.

### Methodological considerations

The major strength of this analysis is its basis on data that were collected every week over a full year, thus removing most of the risk of memory decay and recall bias that would have occurred with traditional retrospective data collection at, for example, 3 and 12 months. Back-filling of information was also avoided, as has otherwise been observed for paper diaries in long-term data collection [10]. This approach of data collection appears not to have been used in other similar studies.

A potential weakness of our analysis is that the data came from two different studies. However, the study subjects in these two studies were recruited from the same treatment centre at the same time, with patients referred from the same catchment area, using the same method of data collection and involved mostly the same researchers. This gave consistency in study design, inclusion criteria, and data collection methods and probably removed some external circumstances that could otherwise have affected the results. However, in the unlikely case that patients referred to this back pain centre had some characteristics that could affect the outcome variable in a biased manner, the results would not be generalizable to other similar clinical populations.

Further, these two studies consisted of trials showing no differences in outcome between treatment and a control group. For this reason, treatment and control data were grouped together in each study and analyzed as two study samples. Theoretically, though, it was possible that there were other, more subtle, differences in outcomes between these groups, which could have made this approach unsuitable.

One of the major differences between the two studies was the procedure for handling patient compliance. In Study 1, more effective information was given to the participants on how to provide answers with the SMS-Track program, resulting in only the occasional missing answer. In Study 2, the same careful procedure had not been used, resulting in some incomplete data that had to be removed from the current analysis. However, for those in Study 2 who were included in the analysis but had replied to text messages less than 50 % of the time, the profile was very similar to that of the others, indicating that their non-response did not really bias the reported pattern of LBP.

Our study results are based on one mode of data collection only, in the form of weekly text messages. A potential problem in our study is that the question on LBP included only the description of “bothersome” pain without any additional clarification on severity or type of pain. Although the concept of “bothersomeness” is expected to capture more severe pain, and not an occasional twinge of short-lasting or mild pain [11], it is possible that patients with, for example, Modic changes or some other subgroup in this study material could have experienced a specific type of pain or a diurnal fluctuation pattern that was not captured.

However, in all, we consider that this simple study brings new and reliable insights into the variations of ‘chronicity’ through the mere use of trajectory data.

## Conclusion

This study revealed there to be considerable variation in weekly persistence of symptoms during 1 year in patients from the secondary care sector with chronic LBP. The results range from bothersome pain each day of the week, every week of the year, to no weeks at all with 7 days of pain. Interestingly, this pattern is near-identical in the two study samples; those with non-specific LBP and those with LBP and Modic changes. This heterogeneic pain profile in patients with chronic LBP deserves to be further investigated.
